# Comparison of Feature Learning Methods for Human Activity Recognition Using Wearable Sensors

**DOI:** 10.3390/s18020679

**Published:** 2018-02-24

**Authors:** Frédéric Li, Kimiaki Shirahama, Muhammad Adeel Nisar, Lukas Köping, Marcin Grzegorzek

**Affiliations:** 1 Research Group for Pattern Recognition, University of Siegen, Hölderlinstr 3, 57076 Siegen, Germany; kimiaki.shirahama@uni-siegen.de (K.S.); adeel.nisar@uni-siegen.de (M.A.N.); lukas.koeping@uni-siegen.de (L.K.); marcin.grzegorzek@uni-siegen.de (M.G.); 2 Department of Knowledge Engineering, University of Economics in Katowice, Bogucicka 3, 40-226 Katowice, Poland

**Keywords:** human activity recognition, multimodal time series processing, feature learning, deep neural networks, evaluation framework

## Abstract

Getting a good feature representation of data is paramount for Human Activity Recognition (HAR) using wearable sensors. An increasing number of feature learning approaches—in particular deep-learning based—have been proposed to extract an effective feature representation by analyzing large amounts of data. However, getting an objective interpretation of their performances faces two problems: the lack of a baseline evaluation setup, which makes a strict comparison between them impossible, and the insufficiency of implementation details, which can hinder their use. In this paper, we attempt to address both issues: we firstly propose an evaluation framework allowing a rigorous comparison of features extracted by different methods, and use it to carry out extensive experiments with state-of-the-art feature learning approaches. We then provide all the codes and implementation details to make both the reproduction of the results reported in this paper and the re-use of our framework easier for other researchers. Our studies carried out on the OPPORTUNITY and UniMiB-SHAR datasets highlight the effectiveness of hybrid deep-learning architectures involving convolutional and Long-Short-Term-Memory (LSTM) to obtain features characterising both short- and long-term time dependencies in the data.

## 1. Introduction

Human Activity Recognition (HAR) is a research topic which has attracted an increasing amount of attention from the research community, in the wake of the development and spread of increasingly powerful and affordable mobile devices or wearable sensors. The main goal of HAR is automatic detection and recognition of activities from the analysis of data acquired by sensors. HAR finds potential applications in numerous areas, ranging from surveillance tasks for security, to assistive living and improvement of the quality of life or gaming [1,2].

**Figure 1:**
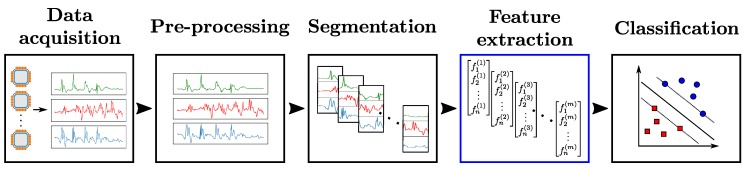
Activity Recognition Chain (ARC). Raw data are firstly acquired from sensors. After pre-processing, segments of data are extracted (Segmentation) and values relevant to the recognition problem are computed from them (Feature extraction). A classifier is then trained and evaluated using those features (Classification). In our framework, all steps of the ARC—except the feature extraction part—are fixed, and a Support-Vector-Machine (SVM) classifier is used for the classification stage.

The development of a HAR system typically follows a standardized sequence of actions involving the use of sensor engineering, data analysis and machine learning techniques. Figure 1 illustrates this process—referred to as *Activity Recognition Chain (ARC)* [3]—which comprises the following steps:

1. Data acquisition: Choice and setup of wearable sensors. Constraints on the choice of sensors due to the nature of activities to be recognized, or potential implementation-related issues (e.g., obstrusiveness, energy consumption, difficulty to setup on the subject, etc. [2]) have to be taken into account at that stage.
2. Data pre-processing: Operations to make the data suitable for further analysis. They comprise various techniques including sensor calibration, unit conversion, normalization or cleaning of corrupted data which could be caused by possible hardware failures or problems on data transfer. In cases involving the use of several sensor modalities, the synchronization of different sensor channels also has to be considered.
3. Segmentation: Identification of segments which contain information about the activities to be recognized. This step serves two main purposes. It filters the data by excluding parts irrelevant to the recognition problem. Additionally, it reduces the quantity of data to be processed by the subsequent stages of the ARC by extracting segments of limited size. This last point especially matters in practice, as hardware-related constraints can limit the amount of data which are possible to process at each time step.
4. Feature extraction: Computation of values to abstract each segment of data into a representation relevant to the activity associated with this segment. Finding a high-level representation is important to ensure the generalization capacity of the HAR system on large-scale data.
5. Classification: Construction of a classifier using the extracted features. The classifier provides separations as clear as possible between different activities in the feature space, i.e., to establish rules to distinguish between the features computed on segments associated with one class and the other segments.

Experience has shown the final activity recognition performances to be very dependent on every step of the ARC. While many extensive theoretical studies have also been carried out in the past decades to establish powerful—and nowadays standardized—classification models such as Support Vector Machine (SVM) [4] or softmax classification [5], most of the focus has been put recently on the feature extraction stage, as the effectiveness of the classification is directly impacted by the quality of the obtained feature representation. Traditionally, features were engineered (i.e., hand-crafted) by experts with knowledge in the considered application domain. Several attempts to find effective features have in particular been done in image-processing-related fields, such as image classification [6], image retrieval [7] or object detection/recognition/tracking [8]. However, this approach is not always possible in practice, for instance when the structure of the input data is unknown a priori because of a lack of expert knowledge in the domain. Additionally, there is no guarantee for features crafted manually to be optimal. For this reason, finding more systematic ways to get good features has drawn an increasing research interest [9].

Notable progress has been done recently to find *feature learning* techniques allowing models to learn automatically features from data with minimal or even no manual input. Solutions using Artificial Neural Networks (ANNs) have especially exploded in popularity in the past few years. ANNs are composed of connected non-linear computational units called artificial neurons, organized in a layer-wise structure, with each layer being composed of an ensemble of neurons. Each neuron of a layer encodes a specific feature computed on the outputs of the previous layer; a behavior particularly highlighted for image classification [10]. Deep ANNs (i.e., composed of an input and output layers, and at least two *hidden* (intermediate) layers) can craft highly abstract features by stacking hidden layers, which enables the computation of features on the low-level descriptors encoded by the first layers of the network. The ways to re-use learned features are also increased by allowing a higher number of possible connections between input and output neurons [9]. The effectiveness of deep ANNs has been demonstrated in many fields other than image classification, such as audio and natural language processing or transfer learning, as surveyed in [9]. Consequently, studies using deep-learning based solutions for HAR using wearable sensors have multiplied for the past few years [11,12,13], all reporting state-of-the-art performances obtained on various HAR benchmark datasets.

However, despite the promising results obtained, it remains actually difficult to assess rigorously the efficiency of feature learning methods, especially deep-learning related ones. The lack of details about the pre-processing of data —sometimes even about the implementation of the feature learning process itself —can make the reproduction of the reported performances complicated. It is also difficult to compare the different approaches because of a lack of standardization regarding the ARC steps: differences in the benchmark HAR dataset(s), data pre-processing operations, segmentation techniques and parameters, or classification models are all obstacles hindering this goal.

The main contribution of this paper is to tackle both of the aforementioned issues regarding the evaluation of feature learning models for HAR. To address the first problem concerning the lack of standardized framework, we define our own evaluation framework where all stages of the ARC— except the feature extraction step—are fixed (as shown in Figure 1), taking inspiration from the comparative study carried out for image classification in [14]. This framework enables us to evaluate and compare the different feature learning models on a fair and objective basis. Specifically, our framework is used to perform comparative studies on two public benchmark datasets: OPPORTUNITY [15] and UniMiB-SHAR [16]. In addition, further experiments to assess the impact of hyper-parameters related to the data acquisition and segmentation steps of the ARC are carried out.

To address the second issue regarding the lack of implementation details, we provide all the datasets, codes and details on how to use them needed to make the reproduction of our results as easy as possible (all research materials used in our studies are provided at http://www.pr.informatik.uni-siegen.de/en/human-activity-recognition). In addition, a generic evaluation script is made available. It can be re-used by other researchers to rigorously evaluate the performances of their own feature learning approaches on the datasets we used, in such a way that allows direct comparisons with our own results.

The comparative studies using our evaluation framework are novel, because such an analysis has in particular been missing in most survey papers of sensor-based HAR [1,2,3]. More precisely, a comprehensive taxonomy of sensor-based approaches (i.e., using time series data, as opposed to vision-based ones using images and/or videos) is realized in [1], describing past works involving the use of generative models (i.e., attempting to build a probabilistic description of the data space, such as Hidden Markov Fields (HMF), Dynamic Bayesian Networks (DBN), etc.) and discriminative models (i.e., attempting to map the input data to class labels, like SVM). Similarly, [2] provides an overview of different discriminative techniques for HAR using wearable sensors, with a special focus on supervised and semi-supervised approaches (i.e., building models using data fully or partially labeled). Both of the aforementioned surveys however only make a list of different methods, without any comparative study being carried out in a reference evaluation framework. In [3], the authors carried out extensive experiments to assess the effectiveness of several approaches, but only considered simple hand-crafted features and classifiers using a small self-produced dataset. To our best knowledge, no paper therefore presents a rigorous comparison of state-of-the-art feature learning approaches similar to the one done in our paper.

This paper is organized as follows. In Section 2, we list, describe and justify our choice of the feature learning methods in our comparative study. Section 3 presents the details of the datasets used in our comparative study, as well as the pre-processing and segmentation techniques employed on them. Section 4 and Section 5 detail the experimental setup and model implementation on the OPPORTUNITY and UniMiB-SHAR datasets, respectively. An analysis of the results is then performed in Section 6. Finally, Section 7 concludes this paper by summarizing our framework and findings.

## 2. Methods

This section presents succinctly the feature learning approaches selected in our comparative study. We test seven different feature crafting approaches covering most of the recent state-of-the-art ones in HAR:

- Hand-crafted features (HC) comprising simple statistical metrics computed on data: this approach constitutes a baseline in our comparative study as the only manual feature crafting method.
- Multi-Layer-Perceptron (MLP): the most basic type of ANN featuring fully-connected layers. The features learned by this model are obtained in a supervised way. The MLP results are used as a baseline for automatic supervised feature crafting.
- Convolutional Neural Network (CNN): a class of ANN featuring convolutional layers which contain neurons performing convolution products on small patches of the input map of the layer, thus extracting features carrying information about local patterns. Apart from image processing [17], audio recognition [18] and natural language processing [19], CNNs recently started to be used for time series-processing in sensor-based HAR [11].
- Long Short-Term Memory network (LSTM): one of the most successful and widespread variant of Recurrent Neural Networks which feature layers containing LSTM cells, able to store information over time in an internal memory. LSTM networks are used to capture temporal dependencies in diversified application fields such as automatic translation [20], image captioning [21] or video-based activity recognition [22].
- Hybrid model featuring CNN and LSTM layers: taking advantage of the high modularity of ANN-based architectures, previous studies in sensor-based HAR reported that hybrid architectures can extract features carrying information about short and long-term time dependencies, and yield better performances than pure CNNs or LSTM networks [12].
- Autoencoder (AE): a class of ANNs trained in a fully unsupervised way to learn a condensed representation which leads to the most accurate reproduction of its input data on its output layer. The results obtained by this approach are used as a baseline for unsupervised feature learning.
- Codebook approach (CB): an unsupervised feature learning method based on the determination of “representative” subsequences— referred to as *codewords*—of the signals used for the learning. The ensemble of codewords (*codebook*) is then used to extract histogram-based features based on similarities between subsequences of the processed data and codewords. Codebook-based methods can be regarded as one-layer CNN, as codewords are learned in a similar unsupervised way to convolutional kernels. They were used in previous works for time series classification [23] or HAR [24].

Each of the models aforementioned is used to learn features on a training and testing datasets. To evaluate the relevance of the obtained features, we use them to train and evaluate a standard classifier common to all approaches. We decide to use a soft-margin SVM [4] with a linear kernel. A SVM draws a classification boundary based on the margin maximization principle, so that the separation is placed in the middle between examples of two different classes. This makes the generalization error of the SVM independent of the number of dimensions. This characteristic is especially important because the approaches described above usually produce high-dimensional features. It thus makes SVM more suitable for our comparative study than other popular classifiers such as k-Nearest-Neighbours, naive Bayes or decision trees, which might face overfitting problems when confronted to high-dimensional feature vectors [25]. A linear kernel for the SVM classifier was picked as it reduces the computational cost compared to other popular kernels such as the Radial Basis Function (RBF), which especially matters for the implementation of a real-time HAR system. In our study, the soft-margin parameter *C* is adjusted to yield the highest performances for each method separately.

It should be noted that many of the feature extractors in our comparative study require a certain number of hyper-parameters (e.g., learning rate, number of hidden layers, number of units per layer, etc.) to be set. The choice of those parameters has been shown to have a high impact on the final classification performances ([13,26] for HAR using wearable sensors). The choice of the optimal hyper-parameters is a difficult topic, for which several strategies are possible: grid search, random search [27] or more elaborated approaches based on Bayesian optimization [28]. All those optimization strategies however are very time-consuming and demanding in terms of computational resources. Since the focus of our study is the comparison of feature extraction models (rather than their optimization), we use a manual search approach to determine the best hyper-parameters for each model. A more detailed description of each model and its parameters is provided in the following subsections.

### 2.1. Hand-Crafted Features

Hand-crafted features for time series analysis can usually be built using simple statistical values (e.g., average, standard-deviation, etc.) or more elaborated descriptors such as features related to the frequency-domain based on the Fourier transform of signals [29]. Despite the prevalence of feature learning nowadays, manually crafting features remains a viable alternative in classification problems due to its simplicity to setup, and to its lower computational complexity.

### 2.2. Codebook Approach

**Figure 2:**
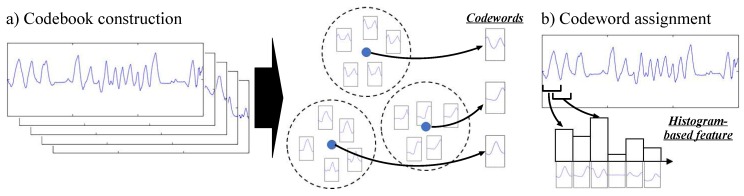
An overview of the codebook approach.

The idea behind the codebook approach is based on two consecutive steps, illustrated in Figure 2. The first step—codebook construction—aims at building a codebook by applying a clustering algorithm on a set of subsequences extracted from the original data sequences. Each center of the clusters obtained this way is then considered as a codeword which represents a statistically distinctive subsequence. The second step—codeword assignment— consists in building a feature vector associated to a data sequence: subsequences are firstly extracted from the sequence, and each of them is assigned to the most similar codeword. Using this information, a histogram-based feature representing the distribution of codewords is then built. A detailed description of each step is provided below.

For the codebook construction step, a sliding time window approach with window size *w* and sliding stride *l* is firstly applied on each sequence to obtain subsequences (*w*-dimensional vector). A *k*-means clustering [30] algorithm is then employed to obtain *N* clusters of similar subsequences, with the Euclidean distance used as similarity metric between two subsequences. It can be noted that the outcome of *k*-means clustering can be dependent on the initialization of the cluster centers, which is performed at random. We therefore run the clustering algorithm 10 times, and select the result for which the sum of the Euclidean distances between subsequences and their assigned cluster centers is minimal. At the end of this step, a codebook consisting of *N* codewords is obtained.

For the codebook assignment step, subsequences are firstly extracted from a sequence using the same sliding window approach employed in the codebook construction step. For each subsequence, the most similar codeword is determined. A histogram of the frequencies of all codewords is then built, with the bin corresponding to one codeword being incremented by one each time the codeword was considered as the most similar to one of the subsequences. The histogram is finally normalized so that the sum of the *N* codeword frequencies yields one to obtain a probabilistic feature representation.

We refer to the approach described above as “codebook with *hard assignment*” (from now on abbreviated as CBH) since each subsequence is deterministically assigned to one codeword. This approach however might lack flexibility to handle “uncertain” situations where a subsequence is similar to two or more codewords. To address this issue, we therefore use a *soft assignment* variant (referred to as CBS) which performs a smooth assignment of a subsequence to multiple codewords based on kernel density estimation [31]. Assuming that $x_{s}$ and $c_{n}$ are the *s*th subsequence (1≤s≤S) in a sequence and the nth codeword (1≤n≤N), respectively, we compute the smoothed frequency $F(c_{n})$ of $c_{n}$ as follows:

(1) $$\begin{array}{c} F\left(c_{n}\right)=\frac{1}{S}\sum_{s=1}^{S}\frac{K_{\sigma }\left(D\left(x_{s},c_{n}\right)\right)}{\sum_{n^{\prime }=1}^{N}K_{\sigma }\left(D\left(x_{s},c_{n^{\prime }}\right)\right)}, \end{array}$$

where $D(x_{s},c_{n})$ is the Euclidian distance between $x_{s}$ and $c_{n}$, and $K_{\sigma }\left(D\left(x_{s},c_{n}\right)\right)$ is its Gaussian kernel value with the smoothing parameter σ. The closer $x_{s}$ is to $c_{n}$ (i.e., the smaller $D(x_{s},c_{n})$ is), the larger $K_{\sigma }\left(D\left(x_{s},c_{n}\right)\right)$ becomes, or, in other words, the bigger the contribution of $x_{s}$ to $F(c_{n})$ becomes. Soft assignment allows us to obtain this way a feature which represents a smoothed distribution of codewords taking into account the similarity between all of them and subsequences. More details about the codebook approach can be found in [24].

### 2.3. Artificial Neural Networks

An ANN consists of an ensemble of connected neurons, each of them taking $n\in N^{*}$ input values $x={\left(x_{1},\ldots ,x_{n}\right)}^{T}$, and applying a non-linear transformation σ—referred to as activation function—on the sum of elements of x weighted by weight parameters $w={\left(w_{1},\ldots ,w_{n}\right)}^{T}$. A bias *b* is added to the weighted sum to introduce flexibility by allowing the activation function to be shifted to the left or right. As a result, the output of a neuron is given by $y=\sigma \left(w^{T}x+b\right)=\sigma \left(\sum_{k=1}^{n}w_{k}x_{k}+b\right)$. Neurons of an ANN are grouped in layers, with outputs of neurons in one layer feeding inputs of the ones in the next layer.

ANNs are usually end-to-end models which directly use raw data and their labels to optimize output probability estimations for each class using an output layer with a softmax activation [5]. To include our ANNs in our evaluation framework, we perform a fine-tuning operation similar to those realized in [14,32]. We firstly train the networks including a softmax output layer in a regular way, using a gradient descent based algorithm. We then remove the softmax layer and use the pre-trained model to compute features on both training and testing datasets. Those deep learning features are finally used to train and evaluate a SVM classifier. To check the relevance of this fine-tuning operation, we performed a preliminary experiment in the setting described in Section 3.1. Its results showed no significant performance difference between using the fine-tuning approach and the usual one using directly the outputs of the softmax layer for the classification (The fine-tuning approach yields even slightly better results than the usual one. Specifically, the former gets an accuracy 91.76%, weighted-F1 score 91.56% and average-F1 score 70.86%, while those of the latter are 91.49%, 91.24% and 69.83% (cf. Section 3.1 for more details on these evaluation metrics)).

Thus, a fair and appropriate performance comparison of deep-learning models can be performed by our framework based on the fine-tuning operation.

We propose to study the performances of some of the most common ANN architectures. A short description of each selected method is provided below.

#### 2.3.1. Multi-Layer Perceptron

**Figure 3:**
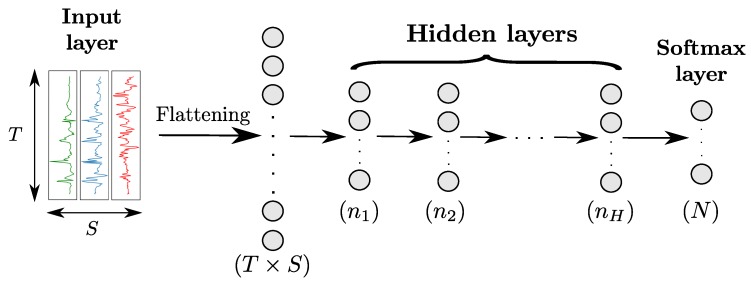
Architecture of a MLP model with *H* hidden layers for sensor-based HAR. Input data from the different sensor channels are first flattened into a (T×S)-dimensional vector and then fed to the hidden layers. All layers are fully-connected. The numbers in parenthesis indicate the number of neurons per layer. *T*, *S* and *N* designate the time length of the input data, the number of sensor channels, and the number of classes, respectively.

Multi-Layer-Perceptron (MLP) is the simplest class of ANN, and involves a hierarchical organisation of neurons in layers. MLPs comprise at least three fully-connected layers (also called *dense layers*) including an input, one or more intermediate (hidden) and an output layer, as shown in Figure 3. Each neuron of a fully-connected layer takes the outputs of all neurons of the previous layer as its inputs. Considering that the output values of hidden neurons represent a set of features extracted from the input of the layer they belong to, stacking layers can be seen as extracting features of an increasingly higher level of abstraction, with the neurons of the nth layer outputting features computed using the ones from the (n-1)th layer.

#### 2.3.2. Convolutional Neural Networks

**Figure 4:**
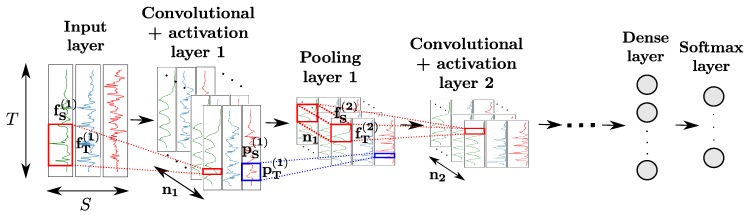
Architecture of a CNN model for sensor-based HAR. *T*, *S* and $n_{k}$ designate the time length of the input data, the number of sensor channels, and the number of convolutional kernels of the *k*th layer, respectively. The convolutional and pooling kernels of the *k*th layer process patches of the input data of sizes $\left(f_{T}^{\left(k\right)},f_{S}^{\left(k\right)}\right)$ and $\left(p_{T}^{\left(k\right)},p_{S}^{\left(k\right)}\right)$, respectively. Neurons of intermediate convolutional layers perform convolution products across all convolutional maps of the previous layer.

CNNs comprise convolutional layers featuring convolutional neurons. The *k*th layer is composed of $n_{k}$ neurons, each of which computes a convolutional map by sliding a convolutional kernel $\left(f_{T}^{\left(k\right)},f_{S}^{\left(k\right)}\right)$ over the input of the layer (indicated in red in Figure 4). Convolutional layers are usually used in combination with activation layers, as well as pooling layers. The neurons of the latter apply a pooling function (e.g., maximum, average, etc.) operating on a patch of size $\left(p_{T}^{\left(k\right)},p_{S}^{\left(k\right)}\right)$ of the input map to downsample it (indicated in blue in Figure 4), to make the features outputted by neurons more robust to variations in temporal positions of the input data. Convolution and pooling operations can either be performed on each sensor channel independently ($f_{S}^{\left(k\right)}=1$ and/or $p_{S}^{\left(k\right)}=1$) or across all sensor channels ($f_{S}^{\left(k\right)}=S$ and/or $p_{S}^{\left(k\right)}=S$).

Similarly to regular dense layers, convolutional-activation-pooling blocks can be stacked to craft high-level convolutional features. Neurons of stacked convolutional layers operate a convolutional product across all the convolutional maps of the previous layer. For classification purposes, a fully-connected layer after the last block can be added to perform a fusion of the information extracted from all sensor channels. The class probabilities are outputted by a softmax layer appended to the end of the network.

#### 2.3.3. Recurrent Neural Networks and Long-Short-Term-Memory

Recurrent Neural Networks (RNNs) are a class of ANNs featuring directed cycles among the connections between neurons. This architecture makes the output of the neurons dependant on the state of the network at the previous timestamps, allowing them to memorize the information extracted from the past data. This specific behavior enables RNNs to find patterns with long-term dependencies.

**Figure 5:**
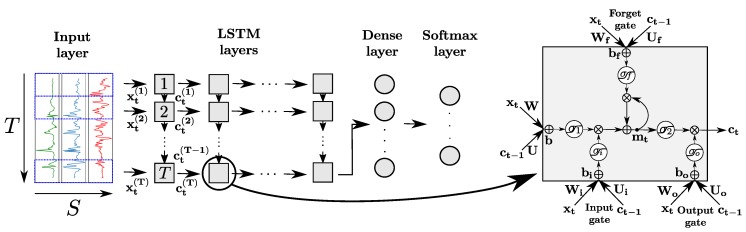
Architecture of a LSTM network for sensor-based HAR (left). The intermediate and last LSTM layers are organized in a many-to-many and many-to-one layout, respectively. Each LSTM layer is composed of LSTM cells (right). $x_{t}$, $m_{t}$ and $c_{t}$ represent the cell input, memory, and output at time *t*, respectively. In addition, ⨁ and ⨂ refer to the element-wise addition and multiplication, respectively. $W_{*}$, $U_{*}$, $b_{*}$ and $\sigma_{*}$ designate internal weight matrices, bias vector and activation function (for gate *∈{i,o,f}). $\sigma_{1}$ and $\sigma_{2}$ are internal activation functions applied on the input and memory of the cell, respectively.

In practice, RNNs are very affected by the problem of *vanishing or exploding gradient*, which refers to the phenomenon happening when derivatives of the error function with respect to the network weights become either very large or close to zero during the training phase [33]. In both cases, the weight update by the back-propagation algorithm is adversely affected. To address this issue, a variant of the standard neuron called Long-Short-Term-Memory (LSTM) cell was proposed [34]. The latter is designed to remember information over time by storing it in an internal memory, and update, output or erase this internal state depending on their input and the state at the previous time step. This mechanism, depicted in Figure 5, is achieved by introducing several internal computational units called *gates*, each featuring their own weights, bias, and activation functions. An input, output and forget gates are used to respectively block the input of the cell (preserving its memory $m_{t}$), block its output (preventing it from intervening in the computation of $m_{t+1}$), and erase its internal memory at time *t*. All gates operate on the input vectors $x_{t}$ of the cell at time *t*, and the outputs of the cell at the previous time step $c_{t-1}$, following the equations

(2) $$\begin{array}{c} y_{t}^{i}=\sigma_{i}\left(W_{i}x_{t}+U_{i}c_{t-1}+b_{i}\right) \end{array}$$

(3) $$\begin{array}{c} y_{t}^{f}=\sigma_{f}\left(W_{f}x_{t}+U_{f}c_{t-1}+b_{f}\right) \end{array}$$

(4) $$\begin{array}{c} y_{t}^{o}=\sigma_{o}\left(W_{o}x_{t}+U_{o}c_{t-1}+b_{o}\right) \end{array}$$

(5) $$\begin{array}{c} m_{t}=y_{t}^{i}⊗\sigma_{1}\left(Wx_{t}+Uc_{t-1}+b\right)+y_{t}^{f}⊗m_{t-1} \end{array}$$

(6) $$\begin{array}{c} c_{t}=y_{t}^{o}⊗\sigma_{2}\left(m_{t}\right) \end{array}$$

with $y_{t}^{*}$ representing the output of gate ∗ at time *t* for ∗∈{i,o,f} (referring to input, output and forget respectively), and $m_{t}$ indicating the memory state of the cell at time *t*. $\sigma_{*}$ designates the activation function, $W_{*}$, $U_{*}$ the matrices of weights, and $b_{*}$ the vector of biases of the gate ∗∈{i,o,f}. W, U and b are matrices of weights and a bias vector for the input of the cell, respectively. $\sigma_{1}$ and $\sigma_{2}$ are activation functions designed to squash the input and output of the cell, respectively. ⊗ represents the element-wise multiplication of two vectors.

Similarly to regular neurons, LSTM cells are organized in layers, with the output of each cell being fed to its successive cell in the layer and to the next layer of the network. LSTM layers can be organized following several patterns, including in particular *many-to-many* (the outputs of all cells of the layer are used as inputs of the next one) and *many-to-one* (only the output of the last cell is used as input of the next layer). The output of the last layer can be passed to a dense and softmax layers for the classification problem.

#### 2.3.4. Hybrid Convolutional and Recurrent Networks

**Figure 6:**
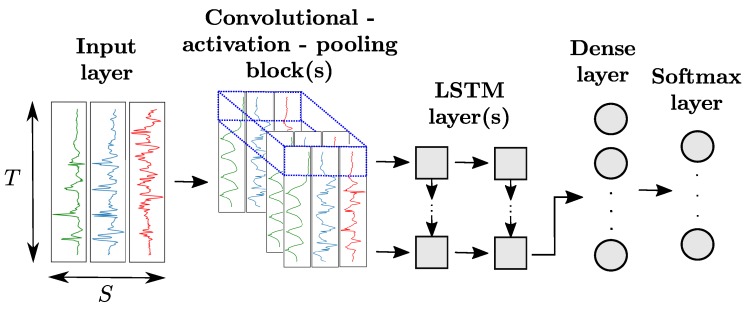
Architecture of a hybrid CNN+LSTM model for sensor-based HAR. Each slice along the time dimension of the output of the convolutional block(s) (in blue) is fed to one LSTM cell. All LSTM layers are organized in a many-to-many pattern, except the last which follows a many-to-one scheme.

Thanks to the high modularity of ANN architectures, it is also possible to append LSTM layers to convolutional blocks as depicted in Figure 6. The last convolutional block of the network produces *n*-dimensional time series with *n* being the number of neurons of the convolutional layer. This output is then sliced along the time dimension. Each slice, indicated in blue in Figure 6, is flattened and fed as input of a LSTM cell. The number of LSTM cells, equal to the number of slices, is dependent on the sizes of the input data, convolutional and pooling kernels of the convolutional blocks.

#### 2.3.5. Autoencoders

**Figure 7:**
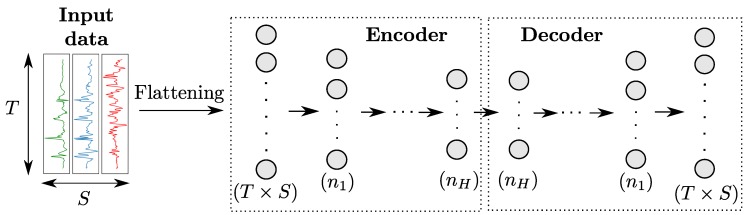
Architecture of an autoencoder model for sensor-based HAR. The numbers in parenthesis refer to the number of neurons per layer. *H* denotes the number of hidden layers in both the encoder and decoder.

An autoencoder (AE) [35] uses a specific architecture consisting in the concatenation of an *encoder* and a *decoder*, as shown in Figure 7. The encoder is a regular network that projects input data in a feature space of lower dimension, while the decoder—whose structure is usually the symmetric of the encoder one—maps the encoded features back to the input space. The AE is then trained to reproduce input data on its output based on a loss function like Mean Squared Errors.

## 3. Data Description

The choice of a dataset is a paramount parameter to establish for any experimental setup. To choose the datasets for our comparative study, we took into account the “complexity” of the data (i.e., whether very high-performing classification solutions were previously found or not), as “solved” datasets would not allow us to find meaningful differences in the performances of the different feature learning approaches. We selected the OPPORTUNITY [15] and UniMiB-SHAR [16] benchmark datasets to carry out our experiments on. This section provides a comprehensive description of both datasets, as well as the pre-processing and segmentation operations.

### 3.1. OPPORTUNITY

The OPPORTUNITY dataset [15] is built from four subjects performing 17 different Activities of Daily Life (ADLs), as listed in Table 1. A NULL class that is not related to any of the 17 ADLs is also present, bringing to a total of 18 distinct classes. The data were collected in a controlled environment simulating a studio flat. They were acquired at a sampling frequency of 30Hz using 7 wireless body-worn inertial measurement units, providing 3D acceleration, rate of turn, magnetic field and orientation information, as well as 12 additional 3D accelerometers placed on the subjects’ arms, back, hips and feet, and accounting for a total of 145 different sensor channels. Each subject was asked to perform 6 runs during the acquisition process: 5 runs—named “ADL1” to “ADL5”—following a scripted scenario leading to the subject performing all of the different activities, and a 6th specific run—named “Drill”—consisting of 20 repetitions of each of the 17 ADLs of the dataset. The data are labeled on a timestamp level, i.e., one label is associated to every sampling time.

As it can be seen in Table 1, the OPPORTUNITY dataset features a notable imbalance in favor of the NULL class which represents 72.28% of the whole dataset. This fact is important for the evaluation phase, as the recognition rate of the dominant class might skew the performance statistics to the detriment of the least represented classes. For this reason, we decide to use an evaluation metric independent of the class repartition (and already used in previous works such as [11,13]): the average F1-score. The F1-score is the harmonic mean of precision (number of true positives divided by the total number of examples evaluated as positive) and recall (number of true positives divided by the number of positive elements). We first compute the F1-score for each class in a one-vs-all way, and then take the their mean value to get the average F1-score. We also add two additional evaluation metrics used in previous works (e.g., [11,12,13,15,16]) to make the comparison with them easier: the overall accuracy (number of examples correctly classified divided by the total number of examples), and the weighted F1-score (sum of class F1-scores, weighted by the class proportion).

The OPPORTUNITY dataset contains a certain number of sensors for which small to very large chunks of data are missing, due to data transmission problems of the wireless sensors during the acquisition process. To bypass this problem, we decided to exclude the sensor channels the most affected by this issue. 38 of them were removed, including data from the left and right hand accelerometers, and quaternion information from the inertial measurement units. For the remaining 107 sensor channels, wecomplement the missing values with the last non-missing one.

For the segmentation phase, we adopt a sliding time-window approach, consisting in extracting “frames” of data by sliding a fixed-length window. We denote by *T* and σ the length of the time window and the sliding stride, respectively. Each frame consists in a T×S matrix, with *S* (=107) being the number of sensor channels. We decide to use a time window of 2 s, resulting in T=64, and a sliding stride of σ=3 following a pre-processing setup similar to the one used in [11].

We decide to use the data from runs ADL1, ADL2, ADL3 and Drill (of all 4 subjects) for our training set, and from the runs ADL 4 and ADL5 for our testing set. This leads to 211,265 and 78,029 frames for the training and testing sets, respectively. Because of the timestamp-level labeling, each frame can usually contain data associated with multiple labels. We opt for a majority labeling, where the label of each frame is determined as the most frequent label among those of the *T* timestamps.

### 3.2. UniMiB-SHAR

The UniMiB-SHAR dataset (University of Milano Bicocca Smartphone-based HAR) [16] aggregates data from 30 subjects (6 male and 24 female) acquired using the 3D accelerometer of a Samsung Galaxy Nexus I9250 smartphone (S=3). The data are sampled at a frequency of 50 Hz, and split in 17 different classes, comprising 8ADLs and 7 “falling” actions as shown in Table 2. Each activity is either performed 2 or 6 times, with half of them having the subject place the smartphone in his/her left pocket, and the other half in his/her right pocket. Unlike OPPORTUNITY, the dataset does not feature any NULL class and remains fairly balanced, despite threeADLs classes having a sensibly higher representation (walking, running and going down).

For this dataset, time windows of data and their associated labels are directly provided with a fixed length T=151 corresponding to approximately 3 s. An energy-based segmentation technique is used by locating time windows centered around peaks of acceleration data [16]. A peak of acceleration is detected at time *t* when the magnitude of the signal is higher than 1.5g (with *g* being the gravitational acceleration constant) and lower than 0 at time t-1. The dataset contains 11,771 time windows of size 151×3 in total.

## 4. Comparative Study on OPPORTUNITY

This section presents the implementation details of the different feature learning approaches and their comparative results on the OPPORTUNITY dataset.

### 4.1. Implementation Details of the Feature Extraction Models

The following sub-sections provide the implementation details of each feature learning approach, and the architectures of the deep-learning models. All the ANNs were coded in Python using the Keras framework [36] with a Tensorflow [37] backend, and trained using the ADADELTA variation of the gradient descent algorithm [38] with default parameters (initial learning rate of 1), for 50 epochs, with a batch size ranging from 100 to 1000. The categorical cross-entropy wasused as the loss function for all models except autoencoders, for which a Mean Squared Errors was used. Experiments was carried out on amachine with an Intel i7-7700K CPU, 16GB RAM and a NVidia GTX 1080Ti GPU. The hyper-parameters of the deep-learning models for the OPPORTUNITY dataset are provided in Table 3.

#### 4.1.1. Hand-Crafted Features (HC)

We use 18hand-crafted features (15 statistical and three frequency-related) (listed in Table 4) computed on each sensor channel of the data frames independently, following the suggestions of [29]. They are then concatenated to yield a feature vector of size 18×S. To remove the effect of the discrepancies between the values taken by each feature, a min-max normalization is performed on each feature to project its values in the interval [0,1]. The normalization constants computed on the training set are re-used for the computation of features on the testing set.

#### 4.1.2. Codebook Approach (CB)

Given a frame, CB extracts a feature from each of S=107 sensor channels. The frame is then represented using “early fusion” which simply concatenates features from all the channels into a single high-dimensional feature [24]. In this framework, a codebook for each channel is constructed using the same set of three hyper-parameters, subsequence length *w*, sliding stride for sampling subsequences *l* and number of codewords *N*. First, it is widely accepted that denser sampling of subsequences yields a better performance [24,39], so *l* is set to 1. Second, we decide to use N=128 based on preliminary experiments using {64,128}, because a higher performance is achieved by more detailed analysis with a larger number of codewords (using *N* larger than 128 is impractical on this dataset, as described below). Last, *w* is set to 24 by examining {8,16,24,32}. In addition, the soft assignment approach (CBS) involves one more hyper-parameter for smoothing the codeword distribution σ, which is set to $2^{8}$ (=256) based on prior tests with $\left\{2^{-5},2^{-4},\cdots ,2^{9}\right\}$.

It should be noted that CB encodes each frame into a 13,696-dimensional vector (107(S)×128(N)). In the hard assignment approach (CBH), this vector is “sparse”, so SVM training based on the sparse vector representations of frames can be efficiently performed and only requires 6.6GB RAM. However, feature vectors in CBS are “dense”, and SVM training needs 69.1GB RAM. Thus, only this is executed on a high-performance computation server with dual Intel Xeon X5690 CPUs and 96GB RAM (even with such a huge RAM consumption, (linear) SVM training can finish in 1021 a).

#### 4.1.3. MLP

We used a MLP with three hidden layers with REctified Linear Units (RELU) activations, taking vectors obtained by flattening frames of data as inputs. We noticed that the addition of a batch normalization layer [40] significantly improved the classification results. Batch normalization layers standardize the inputs of the subsequent layers by computing parameters on batches of training data to normalize each sensor channel independently. It aims at reducing the risk of *internal covariate shift*, which refers to the changes in the distributions of the inputs of a layer during the training phase, and can cause the input values to move toward the saturation regime of the activation function. This phenomenon can potentially decrease gradient values and slow down the training phase. We obtained our best performances by placing a single batch normalization layer directly after the input of the network, although [40] recommends to place them before every activation of hidden layers. This indicates that we can avoid internal covariate shift as long as input data are normalized by a batch normalisation layer. The final architecture used for our MLP models thus consists of a batch normalization layer, followed by three fully-connected layers with RELU activations, and an output layer with a softmax activation providing estimations of probabilities for each class. All hyper-parameters of the model and their values are summarized in Table 3.

#### 4.1.4. CNN

Our CNN architecture involves three consecutive blocks, each including a convolutional, RELU activation and max-pooling layers. Each convolutional kernel performs a 1D convolution over the time dimension, for each sensor channel independently (Preliminary experiments showed that models with convolutions performed across all sensor channels degrade performances on the OPPORTUNITY dataset). Similarly to MLP, adding a batch normalization layer right after the input layer yields significant performance improvements. The model selected in the end comprises in order a batch normalization layer, three blocks of convolutional-RELU-pooling layers, a fully-connected layer and a softmax layer. The hyper-parameters of those layers are provided in Table 3.

#### 4.1.5. LSTM

Based on preliminary experiments, we decide to use a model with two layers. The number of LSTM cells in both layers is equal to the size of the time window *T*. All cells of the network use the sigmoid function for the gate activations, and hyperbolic tangent for the other activations. Each LSTM cell of the first layer takes as inputs the data from all sensor channels at its corresponding time, and the output of the LSTM cell processing the data at the previous time. The first LSTM layer is organized in a many-to-many manner.The second LSTM layer follows a many-to-one outlineA batch normalization layer is also added at the beginning, and a dense and softmax layers at the end of the network. The values of all hyper-parameters of the model are provided in Table 3.

#### 4.1.6. Hybrid CNN and LSTM Models (Hybrid)

Using an architecture similar to the ones in [12], we stack batch normalization, convolutional- RELU-pooling, LSTM, one fully-connected and one softmax layers. After preliminary tests showing that increasing the number of layers did not necessarily improve the performances, we decide to use one convolutional-RELU-pooling block and two LSTM layers on the OPPORTUNITY dataset. The LSTM layers are organised in the same way as the one of the LSTM model described in Section 4.1.5, with the number of LSTM cells being equal to the “time length” of the output of the convolutional-RELU-pooling block. The last LSTM layer follows a many-to-one pattern, and its output is fed to a dense layer, followed by a softmax layer. The values for the convolutional and LSTM hyper-parameters are provided in Table 3.

#### 4.1.7. Autoencoder (AE)

We use a simple autoencoder model, with the encoder and the decoder both featuring only one dense layer. This decision stems from our observations that increasing the number of hidden layers did not improve the classification performances, and that using layers with many neurons instead (as shown in Table 3) yields a significant performance imrovement. All neurons have a RELU activation.

### 4.2. Results

The accuracies, weighted and average F1-scores of all tested feature learning approaches on the OPPORTUNITY dataset are summarized in Table 5. Supervised deep-learning-based feature extraction methods outperform other approaches by a sensible margin. The best performing method is the hybrid CNN and LSTM model (Hybrid), with a weighted F1-score of 70.86% on the OPPORTUNITY dataset. Although a strict comparison is not possible (because of differences in the evaluation frameworks which were employed), an analogy to the results obtained by several previous works, such as [11,12,13], suggests that the results we obtained are state-of-the-art.

While hybrid CNN-LSTM models perform better than using CNN or LSTM separately, it is interesting to note the sensible difference of 4.45% between the average F1-scores of LSTM and CNN. CNN (as well as CBH and CBS) can extract features containing information about short-term time dependencies in the data, with a time horizon constrained by the length of the time window *T* and the size of the convolutional kernels. On the other hand, LSTM can take into account long-term time dependencies thanks to the memory mechanism of LSTM cells. Our observations seem to indicate that features detecting patterns in the long term are more important for the classification of activities on this dataset. Appending a LSTM network to a convolutional block allows the computation of features taking both short- and long-term dependencies into account.

### 4.3. Influence of Data-Related and Segmentation Hyper-Parameters

The previous experiments were carried out in a setup fixing some hyper-parameters related to the first stages of the ARC (depicted in Figure 1). In this section, we present additional studies carried out on slightly modified setups for the data acquisition and segmentation phases to check their influence on recognition performances. We try in particular to modify the number of sensor channels and the size of the sliding time window at the segmentation stage.

#### 4.3.1. Sensor Modalities

Increasing the number of sensor channels *S* allows the amount and diversity of the input data to grow. However, in practice, it also complexifies the setup of the wearable devices. For this reason, it is important to check whether the tested feature extraction methods are robust to variations of *S*.

**Figure 8:**
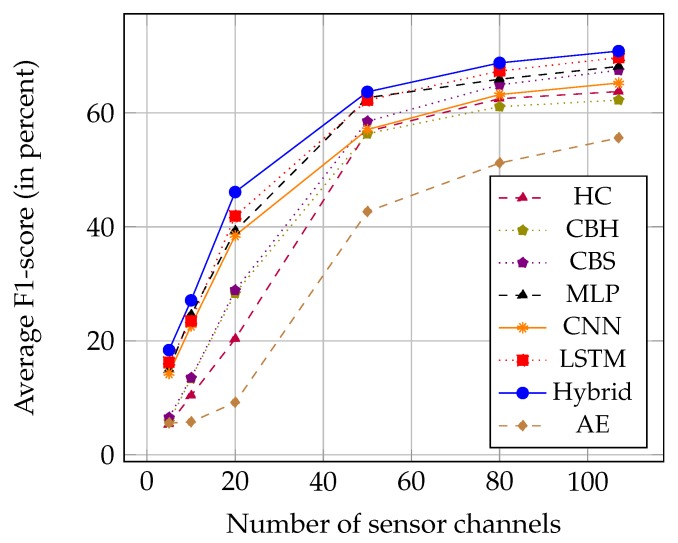
Average F1-scores of the feature learning methods on the OPPORTUNITY dataset, using different numbers of sensor channels (ranked by decreasing variance).

We carried out additional experiments with different subsets of the 107 sensor channels. The creation of subsets is based on the principle of Principal Component Analysis (PCA) [30]. We first rank all sensor channels by decreasing variance (computed on the full dataset). We then form a subset by selecting the *n* sensor channels with the highest variance for n∈{5,10,20,50,80}. The size of the time window is kept at T=64. The results for the average F1-scores are summarized in Table 6 and Figure 8.

As expected, increasing *S* leads to better performances for all different methods, as the variety and amount of data used to train the models increase. The relative performances of the feature extraction approaches also remain unchanged for all the tested numbers of sensor channels. Hybrid models still outperform all other approaches, which confirms the robustness of such deep-learning architectures.

#### 4.3.2. Sliding Time Window Size

The length of the sliding window *T* has a high influence on the features extracted by the different feature learning models. This is especially the case for the deep-learning related methods, for which a variation of the size of the input layer impacts the architecture of the subsequent layers, and causes changes in the field of view of the neurons in convolutional layers, or in the number of LSTM cells of the recurrent layers.

To check whether the trends we observed previously are still relevant regardless of *T*, we carried out two more comparative studies using T=32 (approximately 1 s) and T=96 (approximately 3 s). The results obtained are returned in Table 7.

The trends observed in the reference case (T=64) remain globally unchanged irrespective of the value of *T*. The supervised deep-learning methods still achieve the best performances, with hybrid models outperforming the other methods.

It can be noted that, while changing *T* affects the performances of all models, the best result is obtained in the reference case (T=64). In particular, making the time window larger does not yield better classification performances. This is probably due to the fact that bigger frames potentially contain data related to a higher number of activities, making their majority-labeling more inaccurate.

## 5. Comparative Study on UniMiB-SHAR

This section presents the implementation details of the different feature learning approaches, and the results of their comparative study on the UniMiB-SHAR dataset.

### 5.1. Implementation Details of the Feature Extraction Models

Most of the feature learning approaches (models and training algorithm) employed on the UniMiB-SHAR data remained unchanged compared to those used on the OPPORTUNITY dataset (cf. Section 4.1 for details). The only changes concerned model hyper-parameters. Concerning deep-learning-based models, we found that cross-sensor convolutions improve the classification results (unlike on the OPPORTUNITY dataset). For both CNN and Hybrid, we thus used only one convolutional block, with the convolutional layer performing a cross-sensor convolution. The full list of hyper-parameters is provided in Table 8.

For CB, one codebook is constructed by clustering three-dimensional subsequences to consider correlations among different dimensions. In other words, the codebook construction and codeword assignment steps are carried out by regarding each subsequence as a 3w-dimensional vector (no other modification is needed). Regarding the hyper-parameter setting, *l* is set to 1 for dense sampling of subsequences, and the other hyper-parameters are determined as follows: *w* is fixed to 16 based on preliminary tests using {8,16,32,64,96}, and N=2048 is chosen by examining {128,256,512,1024,2048}. Because of the high computational cost, a codebook is constructed by applying the *k*-means clustering to one million subsequences randomly selected. For the soft assignment approach, σ is set to 4 by testing {0.25,0.5,1,2,4,8,16,32}.

### 5.2. Results

We perform a 30-fold Leave-One-Subject-Out cross validation on the UniMiB-SHAR dataset by using the data from one subject for testing, from the others for training, and then repeating the process for all of the 30 subjects (Because of the high computational cost of CBH and CBS, only one codebook is constructed using subsequences collected from all 30 subjects, and then used to build features on all folds. Subsequences of test subjects are thus also used for the training on each fold, which might slightly skew CB performances. However, experiments on a simple training/testing partition (20 first subjects/10 last subjects, respectively) presented in Section 6 showed that using completely distinct datasets for the codebook construction and evaluation does not change the relative performances of CBS and Hybrid.) The final performance metrics are the average of those obtained for each subject. The results are provided in Table 9. For a baseline comparison, we also add the results obtained by the authors of [16] who directly used the data contained in the time windows as inputs of the classifier (i.e., no feature extraction).

The codebook approach with soft-assignments yields the highest accuracy and weighted F1-score at 77.03% and 75.93% respectively, while the the best average F1-score is obtained by CNN at 64.65%. Overall, CBS, CNN and Hybrid yield sensibly better performances than the other feature extraction methods. We think that this might in part be caused by the specific segmentation technique employed on this dataset: since data frames are exclusively centered around peaks of inputs signals, local features characterizing the shape of the peaks—typically obtained by codebook approaches and ANNs containing convolutional layers—carry a high relevance to the classification problem. The lack of temporal consistency between the different data frames or the absence of frames characterizing transitions between different actions might explain the relatively poor performances displayed by the LSTM architecture.

## 6. Analysis

The results obtained on both datasets highlight three points: the superiority of automatically learned features over hand-crafted ones, the stability of hybrid CNN-LSTM architectures which are able to obtain top performing features on both datasets, and the relevance of the codebook approach with soft-assignment to some specific setups.

If the bad results obtained by hand-crafted features compared to the other feature learning approaches are not unexpected, the gap in their relative performances displayed on both datasets is more surprising: the difference between their average F1-score and the one of the best performing method is 7.39% on OPPORTUNITY, and 49.93% on UniMiB-SHAR. To investigate this, we applied a feature selection method [41] to determine which features are the most relevant to the classification problem. On OPPORTUNITY, we found out that the best features are acquired by sensors placed on the feet of the subjects. We believe this is mainly due to the fact that the dominant NULL class on OPPORTUNITY mainly comprises transitions of the subjects between two different locations of the experimental environment, whereas the others ADLs featured in the dataset do not involve any significant movement of the lower part of the body. Sensors placed on the feet of the subjects and their associated features would then be effective to distinguish the NULL class from the others. On the other hand, the lack of dominant class coupled to the specific peak shape of the signals explains the low performance on the UniMiB-SHAR dataset, as well as its inferiority to the baseline raw data approach.

One interesting observation is the dependence of the performances of deep-learning models on the segmentation phase. Depending on whether the latter is sliding-window-based (e.g., OPPORTUNITY) or energy-based (e.g., UniMiB-SHAR), the type of data frames varies, thus impacting the type of predominant features for the classification. Features characterizing the shape of the time signals— which are typically obtained by convolution-based networks—perform well in the case of an energy-based segmentation, whereas a sliding window segmentation approach makes the memorization of previous data frames more relevant and the LSTM-based architectures more relevant. It can be noted that the hybrid CNN and LSTM model still ranks among the most efficient methods on UniMiB-SHAR, which highlights the stability of this model, and suggests its good generalization capacity on different datasets.

The good performances of CBS on the UniMiB-SHAR dataset is another indicator of the effectiveness of low-level shape-based features in the case of an energy-based segmentation. As we found out that the codebook and deep-learning-based approaches produce different features which yield significantly different results, we expect their fusion to take advantage of this “diversity” and lead to performance improvements [42]. We thus carry out tests on both OPPORTUNITY and UniMiB-SHAR datasets. For this experiment, the UniMiB-SHAR dataset was partitioned using the 20 first and 10 last subjects for the training and testing of our models, respectively and both partitions were used to obtain features using the codebook and hybrid CNN and LSTM. We then perform the fusion by simply averaging the output scores of two SVMs, each trained with one type of features, and using the resulting scores for the class prediction. This kind of simple “late fusion” already proved its effectiveness to improve the performances of deep-learning-based approaches in previous works for video annotation [43]. Table 10 shows a sensible performance improvement by the fusion approach compared to the ones using either codebook or ANN features exclusively on both datasets.

## 7. Conclusions

In this paper, we defined an evaluation framework which fixes all stages of the ARC except the feature extraction step to rigorously compare feature learning methods for sensor-based HAR. We applied this framework on two benchmark datasets, OPPORTUNITY and UniMiB-SHAR, to compare and evaluate the performances of several state-of-the-art methods. The results indicate that a hybrid deep-learning architecture featuring convolutional and LSTM layers is a flexible and robust approach, yielding top performances on both datasets. Changes in hyper-parameters related to the other steps of the ARC, such as the frame size or the number of sensor channels did not change the overall ranking of the feature learning models. The codebook approach also showed to be efficient for an energy-based segmentation case, such as the UniMiB-SHAR dataset. The fusion of features learned by both approaches further improved the results. All research materials used in our studies (i.e., datasets, codes and instructions on how to use them) are also provided to allow the reproduction of our results, and let other researchers test their own features on the datasets we used and rigorously compare their performances to ours.

## Figures and Tables

**Table 1:** Classes of the OPPORTUNITY dataset and their respective proportions of the dataset.

Class	Proportion	Class	Proportion
NULL	72.28 %	Open Drawer 1	1.09 %
Open Door 1	1.87 %	Close Drawer 1	0.87 %
Open Door 2	1.26 %	Open Drawer 2	1.64 %
Close Door 1	6.15 %	Close Drawer 2	1.69 %
Close Door 2	1.54 %	Open Drawer 3	0.94 %
Open Fridge	1.60 %	Close Drawer 3	2.04 %
Close Fridge	0.79 %	Clean Table	1.23 %
Open Dishwasher	1.85 %	Drink from Cup	1.07 %
Close Dishwasher	1.32 %	Toggle Switch	0.78 %

**Table 2:** Classes of the UniMiB-SHAR dataset and their respective proportions. ADLs and falls are provided in the left and right column, respectively.

Class	Proportion	Class	Proportion
Standing Up from Sitting	1.30 %	Falling Forward	4.49 %
Standing Up from Laying	1.83 %	Falling Left	4.54 %
Walking	14.77 %	Falling Right	4.34 %
Running	16.86 %	Falling Backward	4.47 %
Going Up	7.82 %	Falling Backward Sitting-chair	3.69 %
Jumping	6.34 %	Falling with Protection Strategies	4.11 %
Going Down	11.25 %	Falling and Hitting Obstacle	5.62 %
Lying Down from Standing	2.51 %	Syncope	4.36 %
Sitting Down	1.70 %		

**Table 3:** Hyper-parameters of the deep-learning-based models found for T=64 on the OPPORTUNITY dataset. The parameters values are given assuming that the input of the models is of size T×S. Convolutional kernels and pooling sizes are, respectively, given as $\left(f_{T}^{\left(k\right)},f_{S}^{\left(k\right)}\right)$ and $\left(p_{T}^{\left(k\right)},p_{S}^{\left(k\right)}\right)$ (based on their definition in Section 2.3.2).

Model	Parameter	Value
MLP	· # neurons in dense layers 1,2 and 3	2000
CNN	· Conv. kernel size for blocks 1, 2 and 3	(11,1), (10,1), (6,1)
	· Conv. siding stride for blocks 1, 2 and 3	(1,1), (1,1), (1,1)
	· # conv. kernels in blocks 1, 2 and 3	50, 40, 30
	· Pool. sizes for blocks 1, 2 and 3	(2,1), (3,1), (1,1)
	· # neurons in dense layer	1000
LSTM	· # LSTM cells in layers 1 and 2	64, 64
	· Output dim. of LSTM cells in layers 1 and 2	600, 600
	· # neurons in dense layer	512
Hybrid	· Conv. kernel size	(11,1)
	· Conv. sliding stride	(1,1)
	· # conv. kernels	50
	· Pool. size	(2,1)
	· # LSTM cells in layers 1 and 2	27, 27
	· Output dim. of LSTM cells in layers 1 and 2	600, 600
	· # neurons in dense layer	512
AE	· # neurons in encoder & decoder dense layer	5000

**Table 4:** List of the hand-crafted features used in our study. Each feature is computed on each sensor channel independently.

Hand-Crafted Features		
Maximum	Percentile 50	First-order mean
Minimum	Percentile 80	Norm of the first-order mean
Average	Interquartile	Second-order mean
Standard-deviation	Skewness	Norm of the second-order mean
Zero-crossing	Kurtosis	Spectral energy
Percentile 20	Auto-correlation	Spectral entropy

**Table 5:** Classification performance metrics (in percent) of different feature extraction models on the OPPORTUNITY dataset.

Method	Accuracy	Weighted F1-Score	Average F1-Score
HC	89.96	89.53	63.76
CBH	89.66	88.99	62.27
CBS	90.22	89.88	67.50
MLP	91.11	90.86	68.17
CNN	90.58	90.19	65.26
LSTM	91.29	91.16	69.71
Hybrid	**91.76**	**91.56**	**70.86**
AE	87.80	87.60	55.62

**Table 6:** Average F1-scores (in percent) of the feature learning models for different numbers of sensor channels on the OPPORTUNITY dataset.

Number Sensors	HC	CBH	CBS	MLP	CNN	LSTM	Hybrid	AE
5	5.35	5.81	6.53	14.94	14.25	16.25	**18.38**	5.57
10	10.36	13.38	13.50	24.57	22.63	23.50	**27.10**	5.75
20	20.32	28.34	28.88	39.29	38.47	41.89	**46.11**	9.17
50	56.75	56.35	58.52	62.68	57.08	62.23	**63.70**	42.67
80	62.50	61.10	64.93	65.82	63.23	67.36	**68.79**	51.21
107	63.76	62.27	67.50	68.17	65.26	69.71	**70.86**	55.62

**Table 7:** Performance metrics (in percent) of different feature extraction models on the OPPORTUNITY dataset with different window sizes *T*.

2*Method	T = 32			T = 64 (Baseline)			T = 96		
	Acc.	WF1	AF1	Acc.	WF1	AF1	Acc.	WF1	AF1
**HC**	88.74	88.32	58.93	89.96	89.53	63.76	90.38	89.98	64.87
**CBH**	88.99	88.23	58.20	89.66	88.99	62.27	89.30	88.81	61.99
**CBS**	89.61	89.07	63.72	90.22	89.88	67.5	90.36	89.96	66.92
**MLP**	90.79	90.4	66.33	91.11	90.86	68.17	90.94	90.65	66.37
**CNN**	90.34	89.71	62.10	90.58	90.19	65.26	90.38	89.98	63.38
**LSTM**	90.88	90.60	67.20	91.29	91.16	69.71	91.33	91.21	68.64
**Hybrid**	**91.10**	**90.75**	**67.31**	**91.76**	**91.56**	**70.86**	**91.44**	**91.25**	**69.04**
**AE**	85.88	85.90	49.54	87.80	87.60	55.62	86.84	86.78	52.67

**Table 8:** Hyper-parameters of the deep-learning-based models on the UniMiB-SHAR dataset. The parameters values are given assuming that the input of the models is of size T×S. Convolutional kernels and pooling sizes are respectively given as $\left(f_{T}^{\left(k\right)},f_{S}^{\left(k\right)}\right)$ and $\left(p_{T}^{\left(k\right)},p_{S}^{\left(k\right)}\right)$ (based on their definition in Section 2.3.2).

Model	Parameter	Value
MLP	· # neurons in dense layers 1,2 and 3	6000
CNN	· Conv. kernel size	(32,3)
	· Conv. sliding stride	(1,1)
	· # conv. kernels	100
	· Pool. size	(2,1)
	· # neurons in dense layer	6000
LSTM	· # LSTM cells in layers 1 and 2	151, 151
	· Output dim. of LSTM cells in layers 1 and 2	1000, 1000
	· # neurons in dense layer	6000
Hybrid	· Conv. kernel size	(32,3)
	· Conv. sliding stride	(1,1)
	· # conv. kernels	100
	· Pool. size	(2,1)
	· # LSTM cells in layers 1 and 2	60, 60
	· Output dim. of LSTM cells in layers 1 and 2	1000, 1000
	· # neurons in dense layer	6000
AE	· # neurons in encoder & decoder dense layer	6000

**Table 9:** Thirty-fold Leave-One-Subject-Out cross validation performance metrics (in percent) of the different feature extraction models on the UniMiB-SHAR dataset.

Method	Accuracy	Weighted F1-Score	Average F1-Score
Baseline [16]	54.70	—	—
HC	32.01	22.85	13.78
CBH	75.21	74.13	60.01
CBS	**77.03**	**75.93**	63.23
MLP	71.62	70.81	59.97
CNN	74.97	74.29	**64.65**
LSTM	71.47	70.82	59.32
Hybrid	74.63	73.65	64.47
AE	65.67	64.84	55.04

**Table 10:** Performance metrics (in percent) of the codebook approach with soft-assignment, hybrid CNN and LSTM approaches, and their fusion on the OPPORTUNITY and UniMiB-SHAR datasets.

Method	OPPORTUNITY			UniMiB-SHAR		
	Acc.	WF1	AF1	Acc.	WF1	AF1
**CBS**	90.22	89.88	67.50	73.50	73.33	59.62
**Hybrid**	91.76	91.56	70.86	71.04	70.78	59.49
**Fusion**	**92.21**	**91.94**	**72.47**	**74.66**	**74.16**	**62.73**

## Supplementary Materials

The datasets and codes used for the studies presented in this paper are available online at http://www.pr.informatik.uni-siegen.de/en/human-activity-recognition.
